# Two-phase flow visualization under reservoir conditions for highly heterogeneous conglomerate rock: A core-scale study for geologic carbon storage

**DOI:** 10.1038/s41598-018-23224-6

**Published:** 2018-03-20

**Authors:** Kue-Young Kim, Junho Oh, Weon Shik Han, Kwon Gyu Park, Young Jae Shinn, Eungyu Park

**Affiliations:** 1 Korea Institute of Geoscience & Mineral Resources, Daejeon, 34132 South Korea; 2 Department of Geology, Kyungpook National University, Daegu, 41566 South Korea; 3 Department of Earth System Sciences, Yonsei University, Seoul, 03722 South Korea

## Abstract

Geologic storage of carbon dioxide (CO_2_) is considered a viable strategy for significantly reducing anthropogenic CO_2_ emissions into the atmosphere; however, understanding the flow mechanisms in various geological formations is essential for safe storage using this technique. This study presents, for the first time, a two-phase (CO_2_ and brine) flow visualization under reservoir conditions (10 MPa, 50 °C) for a highly heterogeneous conglomerate core obtained from a real CO_2_ storage site. Rock heterogeneity and the porosity variation characteristics were evaluated using X-ray computed tomography (CT). Multiphase flow tests with an *in-situ* imaging technology revealed three distinct CO_2_ saturation distributions (from homogeneous to non-uniform) dependent on compositional complexity. Dense discontinuity networks within clasts provided well-connected pathways for CO_2_ flow, potentially helping to reduce overpressure. Two flow tests, one under capillary-dominated conditions and the other in a transition regime between the capillary and viscous limits, indicated that greater injection rates (potential causes of reservoir overpressure) could be significantly reduced without substantially altering the total stored CO_2_ mass. Finally, the capillary storage capacity of the reservoir was calculated. Capacity ranged between 0.5 and 4.5%, depending on the initial CO_2_ saturation.

## Introduction

Atmospherically released carbon dioxide (CO_2_) is considered to be a major deriver behind climate change, and as such geologic CO_2_ storage is considered a key technology in climate change mitigation strategies^1,2^. Both industry and research communities are currently evaluating the safety and feasibility of long-term CO_2_ sequestration, and a number of pilot- and demonstration-scale projects have been conducted as a part of this effort to test, monitor, and verify technologies in various subsurface geological environments^3–5^.

Previous research related to geologic CO_2_ storage has until now focused on evaluating sandstone formations, as their relatively high porosity and permeability suggest greater economic viability than other rock formations. For this reason, the reservoir lithology of most pilot-scale (e.g., Frio, Nagaoka, Ketzin and Otway) and demonstration- or commercial-scale projects (e.g., MGSC Decatur, Sleipner, Snøhvit, In Sala and Gorgon) has been sandstone^3,5^. The average reservoir porosity has been 5–35% and reservoir permeabilities have ranged from as low as 5 mD (In Salah) to 5,000 mD (Sleipner).

One exception was the MRSCP Gaylord project (a Midwest regional carbon sequestration partnership), where the targeted storage formation consisted of dolomite. This was characterized by interbedded, laminated algal dolomudstone, represented by a mean porosity and permeability of 13% and 22.6 mD, respectively^6^. In an effort to assess the geologic storage potentials, basalt formations have also been considered as alternative storage formations^7,8^.

**Figure 1:**
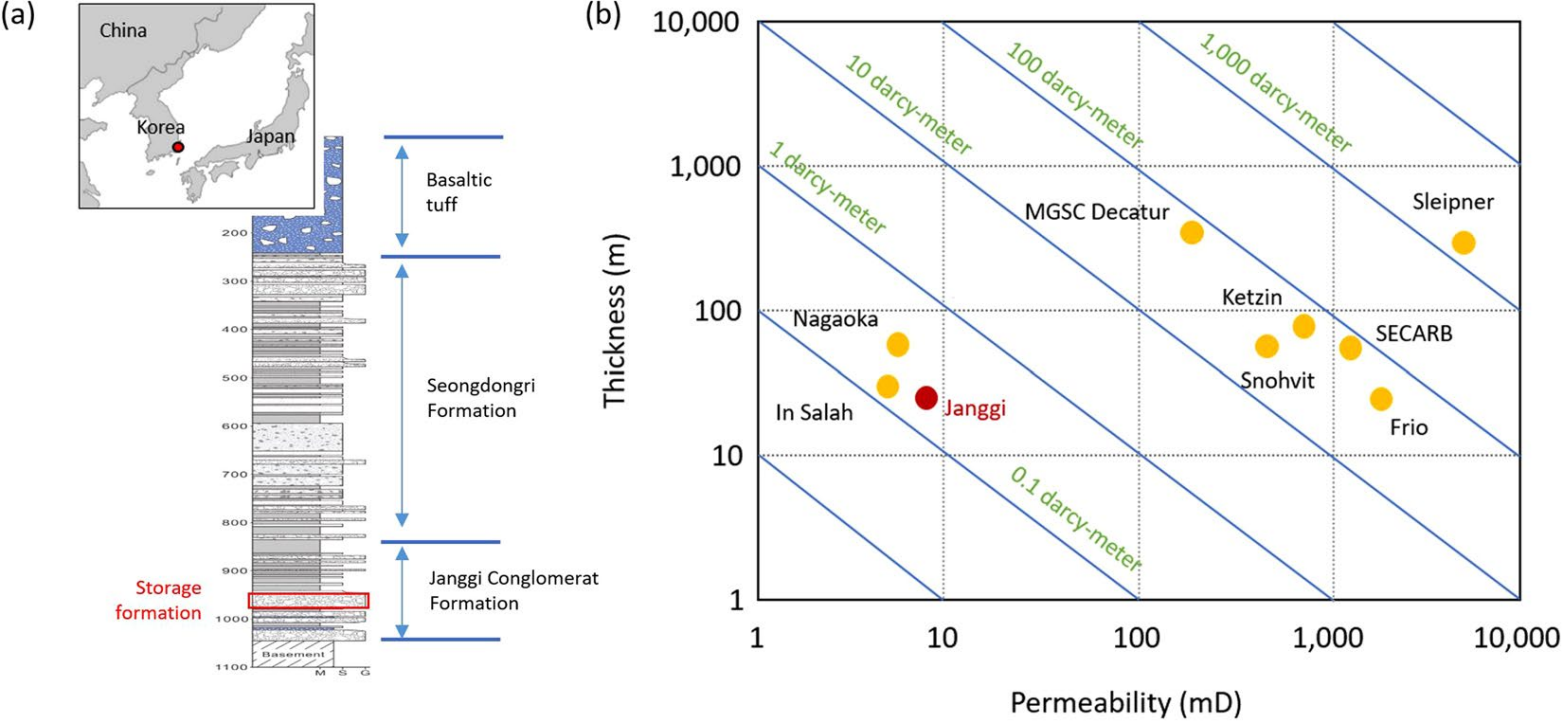
(**a**) The location of onshore Janggi pilot site for CO_2_ storage in South Korea and the representative lithologic log. (**b**) Thickness-permeability cross-plot for the Janggi pilot site, along with data from various existing storage projects.

Although a number of CO_2_ storage projects are being conducted worldwide, the need for additional safe storage verification experiments using various geological formations cannot be overemphasized. The fact that only limited data has been collected during the injection and post-injection phases demonstrates the necessity of additional field assessments of the processes leading to plume stabilization and long term trapping^9^. Followed by on-going international efforts, the first onshore pilot-scale (~10,000 ton) CO_2_ storage project is being conducted in South Korea at the Janggi sedimentary formation, located on a south-eastern portion of the Korean Peninsula (Fig. 1(a)). One distinctive feature differing from other pilot-scale projects is that the targeted formation is a conglomerate, consisting of gravel and cobbles in a silty sand matrix. Figure 1(b) shows a thickness-permeability cross-plot of values selected from various existing storage projects. This plot reveals the injectivity at Janggi falls between 0.1 and 1 darcy-meter, similar to the estimated injectivities at the Nagaoka and In Salah sites.

In addition to field-demonstration studies, well-controlled laboratory experiments are essential for obtaining a fundamental understanding of physical^10,11^ and chemical processes^12,13^ occurring in potential storage formations, as well as predicting their CO_2_ trapping capacities^14^. A number of studies have conducted reactive transport experiments to understand the complex processes of CO_2_-water-mineral interactions and their coupling effects on the CO_2_-water multiphase flow at *in-situ* reservoirs conditions^15–18^. These experimental data can be utilized to validate the numerical studies and build-up confidence in the modeling tools. For example, Smith *et al*.^18^ used detailed experimental data to constrain three-dimensional reactive transport models in order to describe and predict the evolution of pore-space and permeability during geologic CO_2_ storage in carbonate reservoirs.

Core-flooding is a technique that has been used to conduct various laboratory experiments on core samples under conditions closely imitating those of subsurface environments^19^. Due to this advantage, core-flooding tests have been utilized for characterizing the dynamic behaviour of CO_2_ under reservoir conditions. Such tests have focused on resolving various problems related to important factors controlling the behaviours of two-phase fluids, including permeability heterogeneity and multiphase flow^20–22^, measuring relative permeabilities^23–25^, interfacial tension^26,27^, and capillary pressures^28–30^. Additionally, the core-flooding experiments have been applied to assessing residually trapped CO_2_ amounts under reservoir conditions^23,25,31–33^. Still other research topics have included multiphase fluid behaviour in fractured porous media^34,35^, salt-precipitation dynamics^36,37^ and the non-equilibrium dissolution of CO_2_ in a heterogeneous core^32,38^.

Despite these numerous core-scale studies, there has been little investigation on how CO_2_ migrates in a highly heterogeneous porous media. This study presents, for the first time, a two-phase flow visualization under reservoir conditions for a highly heterogeneous conglomerate core that has been obtained from the real CO_2_ storage site. The three-dimensional (3D) distribution of clasts within the matrix was determined utilizing X-ray computed tomography (CT). Additionally, real-time X-ray scanning techniques (conducted during the CO_2_ and brine flow tests) captured the dynamic distribution of CO_2_ saturation in the conglomerate core. Multiphase transport simulations were also conducted to assess the effects of heterogeneity on pressure build-up as well as spatial variation of CO_2_ saturation. Finally, based on the initial-residual (IR) characteristic curve, the storage potential of the conglomerate formation is discussed.

## Results and Discussion

### Heterogeneity characterization

**Figure 2:**
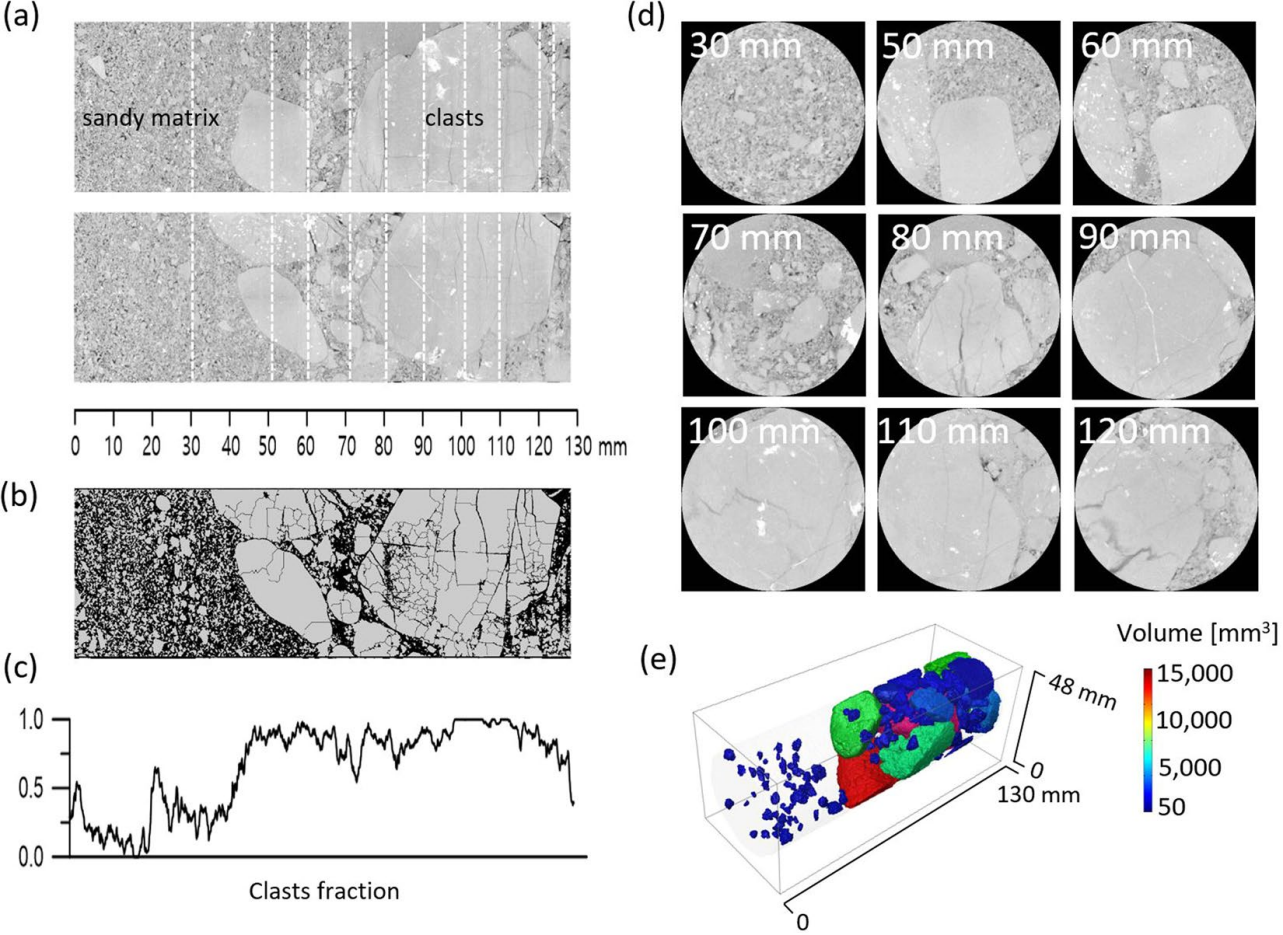
(**a**) Two CT cross-sectional images along the longitudinal axis taken from different angles. (**b**) Binary image of the core-plug for clarification of the discontinuities in the clasts. (**c**) The profile for clasts fraction along the longitudinal core-axis. (**d**) 2D cross-section images along the white-dashed lines shown in (a). (**e**) 3D distribution of clasts in the core-plug.

A core-plug obtained from the Janggi conglomerate formation was used to investigate the spatial distribution of clasts within a silty sand matrix. Figure 2(a) presents two cross-sectional CT images taken along the longitudinal axis at different angles. In addition, Fig. 2(b) shows the binary image generated from the 8-bit CT images, where the boundaries between the clasts and matrix are distinguished more clearly and discontinuities within the clasts are highlighted. The clast fractioning profiles along the longitudinal core-axis are shown in Fig. 2(c). Nine cross-sectional images, taken along the white-dashed lines appearing in Fig. 2(a), are presented in Fig. 2(d). In the upstream region (30 mm) of the core, the constituents are relatively homogeneous and only a small fraction of clasts were preserved (*f* < 0.3). In the midstream portion (70 mm), both medium size of clasts and matrix existed together, whereas larger clast sizes were present in the downstream region (100 mm). Figure 1(e) shows the three-dimensional distribution of clasts in the core-plug.

**Figure 3:**
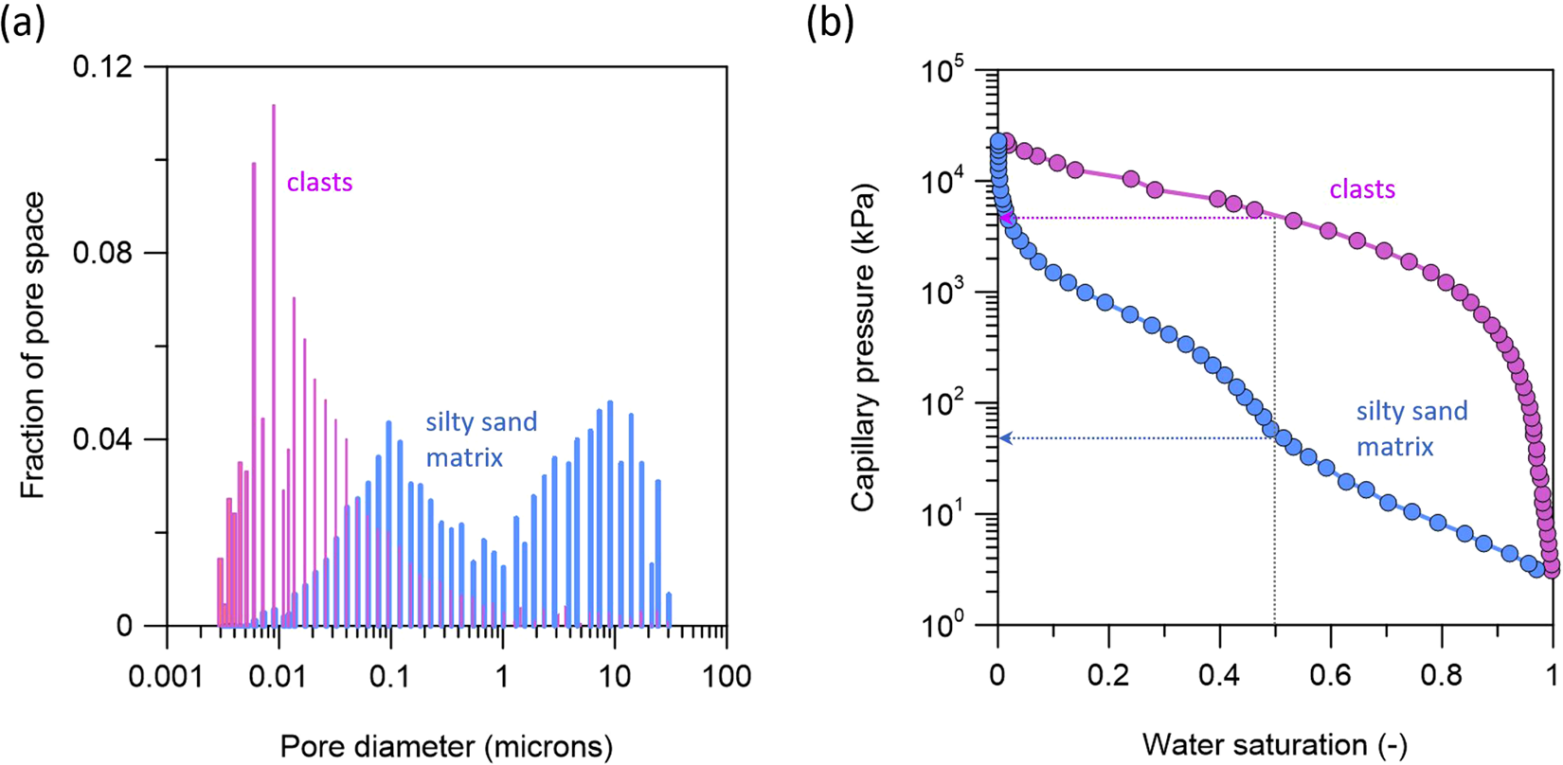
(**a**) Pore size distribution for the silty sand matrix and clasts. (**b**) Capillary pressure (P_c_) curves as a function of brine saturation.

The pore size distribution were analysed for both the matrix and clasts (Fig. 3(a)). The clast regime was characterized by a skewed uni-modal pore-size distribution dominated by micropores (<1 μm diameter). The silty sand matrix regime demonstrated a bi-modal pore size distribution dominated by micropores (<1 μm diameter) and mesopores (>2 μm diameter) (Fig. 3(a)). The bi-modal pore size distribution characteristics of the matrix were also reflected in the shape of the capillary pressure (P_c_) curve, appearing as a superposition of two uni-modal pore size distribution characteristic curves. The P_c_ (S_w_ = 0.5) of the clasts was more than two orders of magnitude greater than that of the matrix (Fig. 3(b)).

### Multiphase flow experiments

This experimental study aimed to characterize the spatio-temporal distribution of CO_2_ saturation build-up during the multiphase flow tests. Two different tests were conducted: (i) CO_2_ injection into a brine-saturated core, simulating the CO_2_ injection period (drainage) conditions; and (ii) brine injection after the CO_2_ injection test, reproducing the post-injection period (imbibition) conditions.

**Figure 4:**
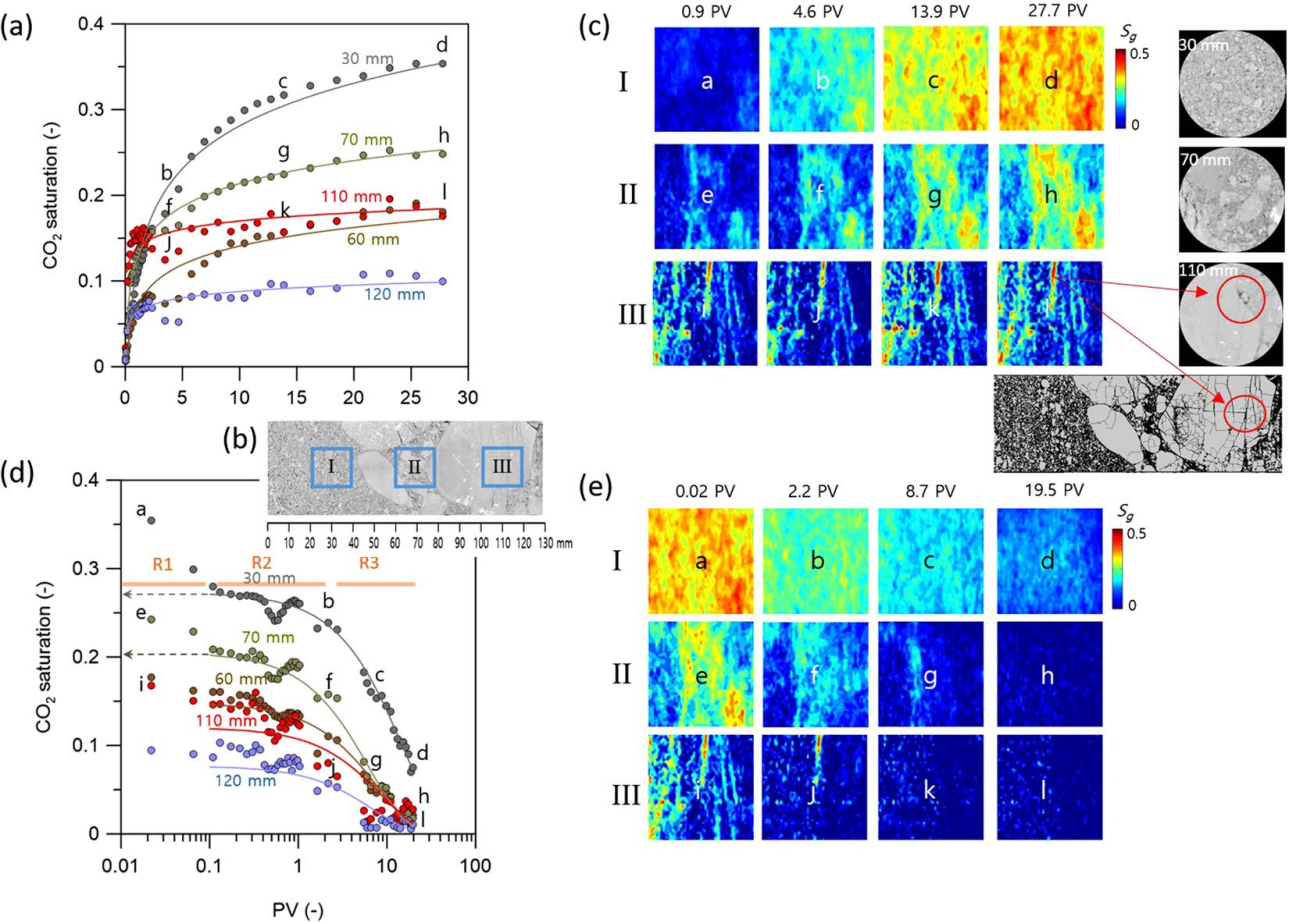
(**a**) Vertically averaged CO_2_ saturation at five different locations (30, 60, 70, 110, and 120 mm) versus pore volume during the CO_2_ injection test. (**b**) Three subdomains (20 × 20  mm) of the core located at upstream (domain I), midstream (domain II), and downstream (domain III) region that are mainly composed of sand, small clasts and large clasts, respectively. (**c**) Snapshots of CO_2_ saturation at three different domains during the CO_2_ injection test. (**d**) Vertically averaged CO_2_ saturation at five different locations (30, 60, 70, 110, and 120 mm) versus pore volume during the brine injection test. (**e**) Snapshots of CO_2_ saturation at three different domains during the brine injection test.

CO_2_ saturation distribution snapshots revealed the propagation of CO_2_ saturation throughout the conglomerate core with increases in pore volume (PV) (Supplementary Figs S1 and S2). Figure 4(a) presents the vertically averaged CO_2_ saturations from the five different locations (30, 60, 70, 110, and 120 mm) delineated in Fig. 2(a). Although the averaging is smoothing out the fluctuation of CO_2_ saturation related to small-scale heterogeneity, it clearly presents the increasing tendency within the core sample. All profiles showed logarithmic increases with the PVs. The CO_2_ saturation profile at 30 mm increased slowly when approaching the asymptote during the flow tests; however, the other profile (such as at 110 mm) approached their asymptotes more rapidly, implying that CO_2_ saturation was quickly stabilized throughout the experiment. It is interesting to note that CO_2_ saturation at the downstream side of the core was greater than that at the upstream side in early stages (<1.0 PV). The spatial inversion of CO_2_ saturation distribution was attributed to the heterogeneous pore space along the conglomerate core, including the presence of complex discontinuities in clasts (Fig. 2(b)). On the upstream side where the matrix was dominant, CO_2_ saturation was low due to the presence of relatively large sized pores, which accommodated the injected CO_2_. Equal amounts of CO_2_ flowing through the downstream side, dominated by clasts, showed elevated CO_2_ saturation because clast discontinuities provided only small pore-spaces.

To perform a detailed assessment of the spatial distribution of CO_2_ saturation, three subdomains (20 × 20  mm) were selected, one each located in the upstream (domain I), midstream (domain II), and downstream (domain III) regions as shown in Fig. 4(b). These domains primarily composed of sand, small clasts, and large clasts, respectively. Domain I (primarily sand) was characterized by a relatively homogeneous CO_2_ saturation distribution over time (Fig. 4(c)) with a small (sub-core) scale heterogeneous CO_2_ saturation observed due to the effect of the bi-modal pore-size distribution preserved in the matrix (Fig. 3(a)). Domain II, which was composed of irregularly spaced small clast fragments, demonstrated heterogeneous CO_2_ saturation caused by irregularly spaced small clast fragments. Low CO_2_ saturations resulted from the presence of clasts, whereas high CO_2_ saturations were observed in high porosity sand. Highly heterogeneous CO_2_ saturation was observed in domain III, caused by a dense network of discontinuities within the large clasts where multiple discontinuities with relatively large apertures (110 mm) were located, as seen in Fig. 4(c). This discontinuity network provided well-connected pathways for CO_2_ flow, resulting in a rapid increase in CO_2_ saturation.

The vertically averaged CO_2_ saturation was also plotted for the brine injection tests (Fig. 4(d)). As discussed in Oh *et al*.^32^, the dynamic transition of CO_2_ trapping could be assessed during the imbibition test. Three regimes of CO_2_ trapping mechanisms were characterized: (i) immediate mobile CO_2_ displacement by injected brine (R1), (ii) immobile CO_2_ preservation as residual trapping (R2), and (iii) the gradual dissolution of residually trapped CO_2_ into the fresh brine (R3). The dynamic transition of CO_2_ trapping at 30 mm displayed initial decrease in CO_2_ saturation from 0.35 to 0.27 during the early stage (0.01–0.1 PV) primarily resulted from the mobile CO_2_ displacement (Regime 1). In Regime 2, the CO_2_ saturation was relatively consistent and stabilized at 0.27 between 0.1 and 1.0 PV, which implies the preservation of immobile CO_2_ (residually trapped). This is possible because the brine that enters the core will initially dissolve a small amount of CO_2_ at the inlet of the core and becomes fully-saturated preventing it from dissolving any more CO_2_. However, as more fresh brine was injected into the core, the residually trapped CO_2_ dissolved into fresh brine (Regime 3).

Figure 4(e) shows CO_2_ saturation snapshots from the three different domains during the brine injection test. In domain I where the matrix dominated, CO_2_ was distributed uniformly at the end of drainage test. Brine displacement tests induced similar uniform distributions in residually trapped CO_2_, which eventually dissolved into the brine. However, in domains II and III, a larger fraction of pores was initially filled with CO_2_, resulting in more snap-off and trapping as brine invaded the pore space. Under these conditions, CO_2_ dissolution was highlighted only within the matrix and discontinuities.

### Multiphase transport simulations

The above experiments were conducted with fluid injection at the left boundary, but CO_2_ injection from the opposite boundary would cause difference in the distribution of CO_2_ saturation due to nature in highly heterogeneous conglomerate core. We used TOUGH2^39^ simulator to model multiphase and multicomponent fluid transport to assess directional effect of fluid flow on pressure build-up as well as CO_2_ saturation distribution along the core sample. A two-dimensional model was constructed based on the CT image of the core sample to reflect the distribution of clasts. Then, two different cases were simulated (case 1: left-to-right flow; case 2: reverse flow).

**Figure 5:**
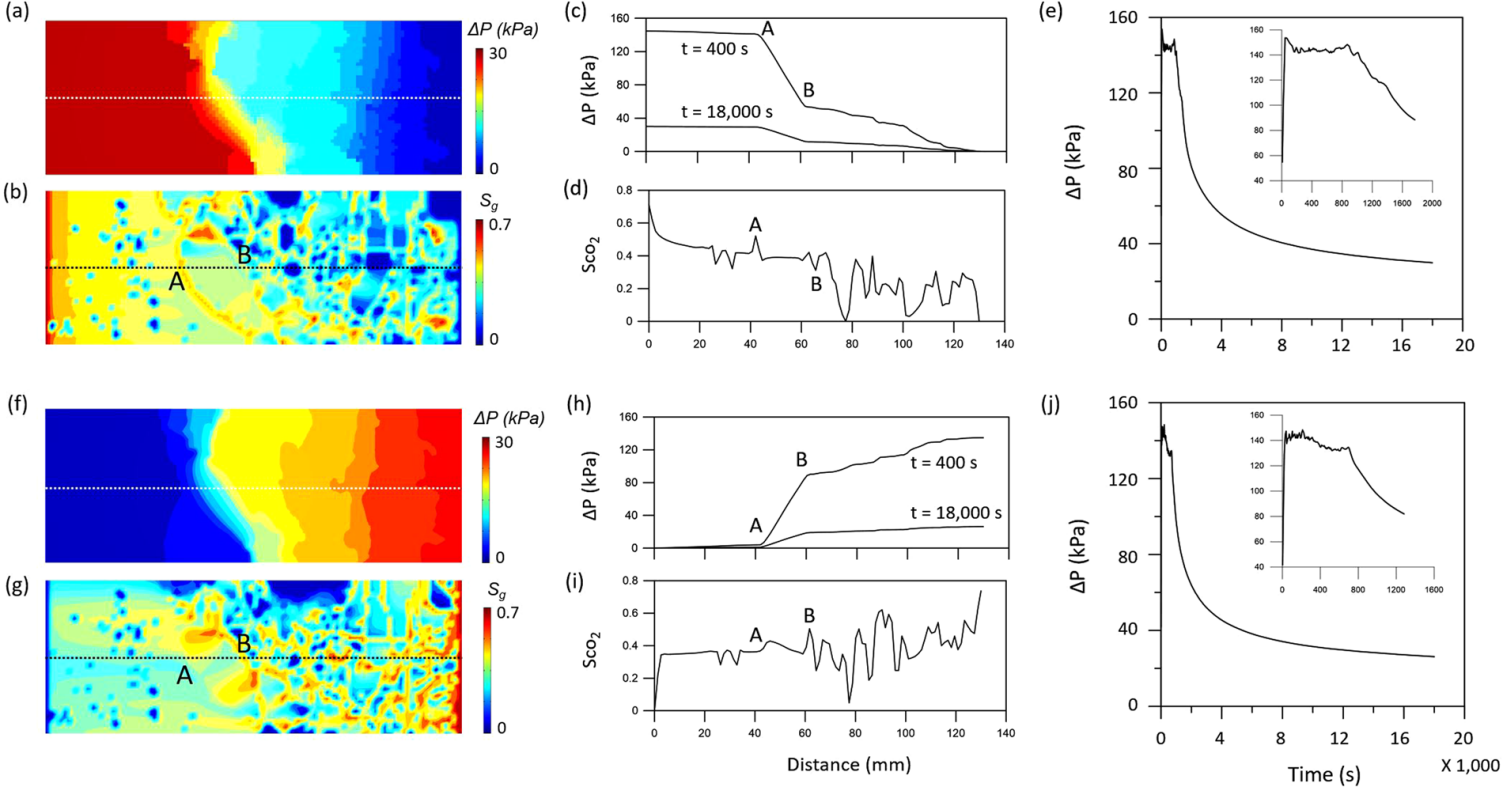
Simulation results for the two different flow directions (case 1: left-to-right flow (**a**–**e**); case 2: right-to-left flow (**f**–**j**)). The results presents pressure (a and f) and CO_2_ saturation map (**b** and **g**), profiles for CO_2_ pressure (**c** and **h**) and saturation (**d** and **i**) along the center of the model domain, and pressure build-up (**e** and **j**).

Figure 5 presents simulation results of pressure (a and f) and CO_2_ saturation map (b and g), profiles for CO_2_ pressure (c and h) and saturation (d and i) along the center of the model domain, and pressure build-up (e and j) for the two different cases. Both cases showed a significant pressure drop between points A and B, which lie on a boundary of a large sized clast. The heterogeneous pressure distribution resulted in different CO_2_ saturation within the model for the two cases. For example, case 1 showed relatively high CO_2_ saturation (S_g_~0.5) at point A and low CO_2_ saturation (S_g_~0.3) at point B, while case 2 showed relatively high CO_2_ saturation (S_g_~0.5) at point B. This implies that the distribution of CO_2_ saturation is dependent on the flow direction. The pressure difference between the inlet and outlet (ΔP) showed ~150 kPa during the early stage of injection and stabilized at ~30 kPa for both cases (Fig. 5(e and j)).

### CO_2_ mass at different capillary numbers

**Figure 6:**
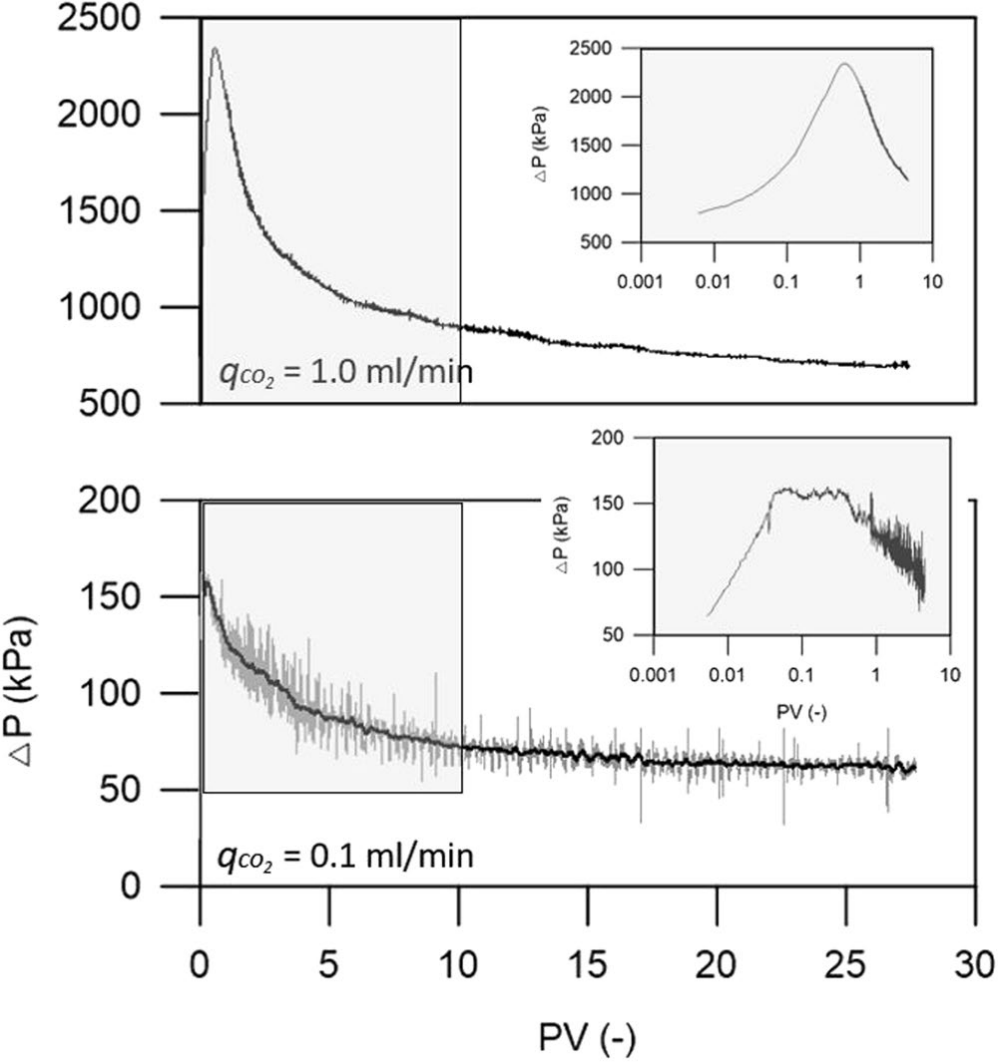
The transient pressure difference (ΔP) across the core for two injection rates (q = 1.0 and 0.1 ml/min).

A dimensionless capillary number (${{\rm{N}}}_{{\rm{c}}}$), representing the relative contributions of viscous versus capillary forces, was set to reflect the reservoir conditions. Two CO_2_ injection tests (qco_2_ = 1.0 and 0.1 ml/min) were performed in this study, producing associated ${{\rm{N}}}_{{\rm{c}}}$ values of ~10^−1^ and ~10^−2^, respectively. The high ${{\rm{N}}}_{{\rm{c}}}$ case was observed in a transition regime between the capillary and viscous limits, whereas the low ${{\rm{N}}}_{{\rm{c}}}$ case was under capillary dominated flow conditions. The transient pressure difference (ΔP) values across the core for the two different cases are shown in Fig. 6. The ΔP build-up under a high injection rate (${{\rm{N}}}_{{\rm{c}}}$ ~ 10^−1^) reached 2,300 kPa in early injection stages before stabilizing to ~700 kPa; however, the low injection rate (${{\rm{N}}}_{{\rm{c}}}$ ~ 10^−2^) displayed a small buildup of ΔP build-up (~150 kPa) in early injection stages before ΔP stabilized to ~65 kPa for the remainder of the multiphase flow experiment.

**Figure 7:**
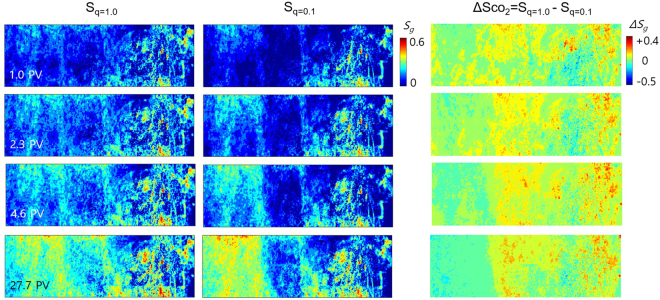
CO_2_ saturation maps for the two different injection rates (qco_2_ = 1.0 and 0.1 ml/min) and the contrasts of CO_2_ saturation (ΔSco_2_ = S_q=1.0_ − S_q=0.1_) between the high and low injection rate cases.

Figure 7 displays the CO_2_ saturation distribution maps for the two injection rates (qco_2_ = 1.0 and 0.1 ml/min) and the CO_2_ saturation contrast (ΔSco_2_ = S_q=1.0_−S_q=0.1_) maps between the high and low injection rate cases. The measured ΔSco_2_ displayed a strong dependency on core heterogeneity, showing negative values on the upstream side (implying lower CO_2_ saturation with a greater injection rate (S_q=1.0_ < S_q=0.1_)). The high injection rate created excessed pressure on CO_2_ (nonwetting phase), enabling CO_2_ to transport across the high P_c_ region on the midstream side. This had a negative impact on the build-up of CO_2_ mass on the upstream side. At low injection rates, however, additional CO_2_ occupied the pore-spaces on the upstream side because CO_2_ transport across the high P_c_ region on the midstream side was hindered. The CO_2_ mass within the core was calculated for both ${{\rm{N}}}_{{\rm{c}}}$ cases to be 2.24 and 2.18 g, respectively, once a steady-state condition was reached. This demonstrated a 10× reduction in injection rate caused only a 2% decrease in CO_2_ mass, suggesting that injection rates, which may cause reservoir overpressure when high, can be significantly reduced without substantially altering the total CO_2_ mass.

### Storage capacity

During the post-injection stage, capillary forces at the pore scale are responsible for breaking up the CO_2_ phase into the form of blobs or ganglia, becoming effectively immobile^40,41^. The capillary trapping potential was evaluated in these conditions based on the performed multiphase flow experiments. The initial-residual (IR) characteristic curves have commonly been used to describe the residual trapping potentials of rock, with numerous developed models describing this relationship. This study applied the Land model^42^, one of the earliest and most widely used trapping models.

**Figure 8:**
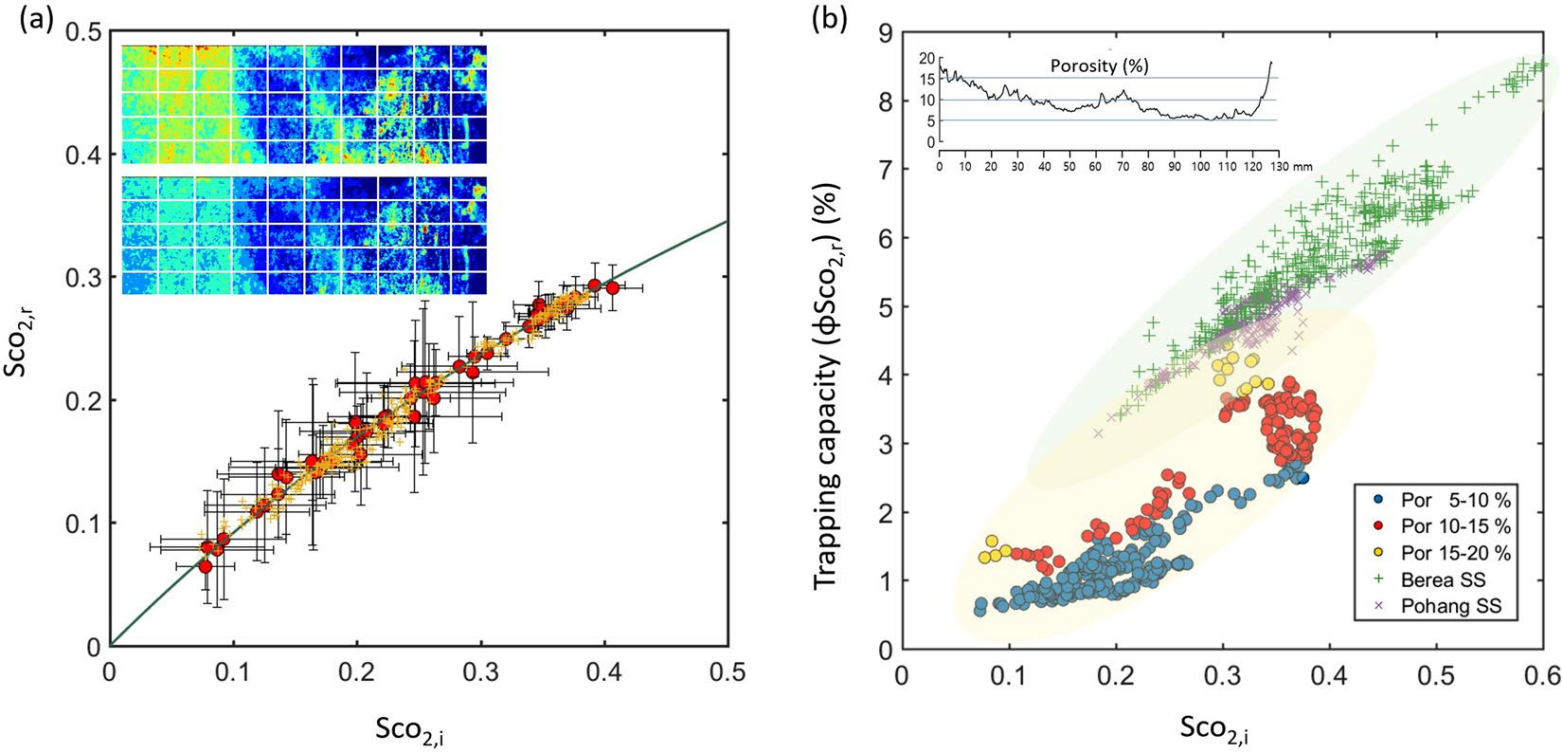
(**a**) A trapping curve relating initial CO_2_ saturation (Sco_2,i_) to residual CO_2_ saturation (Sco_2,r_). The Sco_2,i_ refers to the initial CO_2_ saturation prior to imbibition test while the Sco_2,r_ refers to the residually trapped CO_2_ saturation. Red circles represent the relationship between the Sco_2,i_ and Sco_2,r_ within equally divided subdomains; error bars represent one standard deviation. (**b**) Trapping capacity (φSco_2,r_) versus initial CO_2_ saturation (Sco_2,i_); colors represent different ranges of porosity.

Figure 8(a) presents the relationship between the initial (Sco_2__,__i_) and residual saturations (Sco_2__,__r_), where Sco_2,i_ refers to the initial CO_2_ saturation prior to brine injection testing whereas Sco_2,r_ refers to the residually trapped CO_2_ saturation. For analysis, highly heterogeneous conglomerate core was divided into 50 equal subdomains. Then, based on the brine injection testing, the residual CO_2_ saturation achieved at 0.1 PV (R2) was determined (Fig. 4(d)), and subsequently, the amount of capillary trapped CO_2_ was assessed using the Land model given by ${{\rm{S}}}_{{{\rm{CO}}}_{2,{\rm{r}}}}=({{\rm{S}}}_{{{\rm{CO}}}_{2,{\rm{i}}}})/(1+{\rm{C}}\cdot {{\rm{S}}}_{{{\rm{CO}}}_{2,{\rm{i}}}})$. Here, C is the dimensionless constant known as the Land coefficient. In this study, the calculated C was calculated as 0.9, showing a monotonic increase in the residual saturation as a function of Sco_2_,_i_. This was in agreement with numerous previous works investigating water-wet porous medias.

The capillary-trapping capacity, defined as φSco_2__,__r_ (φ is porosity) is a rescaling of the traditional IR saturation plot^43,44^. This quantity is of great importance in geologic CO_2_ storage applications because it states how much CO_2_ can be stored securely per unit volume of a rock. Figure 8(b) presents the relationship between Sco_2__,__i_ and φSco_2__,__r_ at different porosities (blue, red and orange circle symbols). The IR relationships of other core samples (Berea and Pohang sandstones) are plotted alongside for comparison. Core sample details are described in the Supplementary Table S1. The trapping capacities of both Berea and Pohang sandstones showed more or less linear increases with Sco_2__,__i_, whereas the φSco_2__,__r_ values for heterogeneous conglomerate cores were widely spread and dependent on the size of porosity. Although the overall capacity of the conglomerate core was smaller than those of the Berea or Pohang sandstones, the conglomerate high porosity region displayed a similar capacity to the sandstones (e.g., φSco_2__,__r_ ~4.5% at Sco_2__,__i_ = 0.3).

## Conclusions

Although sandstone formations are considered to be the most appropriate for CO_2_ sequestration, other types of geologic formations are being investigated for this purpose. For example, the target rock formation in MRSCP Gaylord project was dolomite^45^, and the Midale project (an extension of the Weyburn project) demonstrated the possibility for CO_2_ containment in a fractured reservoir^5^. Basaltic formations have also been considered as alternative storage formations^7,8^. In South Korea, a conglomerate formation is being tested for geologic CO_2_ storage.

This study reported a thorough core-scale investigation of a highly heterogeneous conglomerate formation. The rock heterogeneity was investigated with the use of X-ray CT and MICP analysis revealing that the targeted storage formation was composed of a silty sand matrix and irregularly spaced clasts of various sizes. Laboratory multiphase flow tests with flow visualization under reservoir conditions provided important information on CO_2_ migration behaviours as well as the storage capacity of the highly heterogeneous conglomerate. Variations in clast spatial configurations gave rise to different storage features. Dense networks of discontinuities within clasts were observed to provide well-connected CO_2_ flow pathways, causing a rapid increase in CO_2_ saturation, and potentially helping to reduce overpressure. Two flow tests, one in the capillary-dominated condition and the other in a transition regime between the capillary and viscous limits, revealed that a 10× reduction in injection rate caused only a 2% decrease in the CO_2_ mass. This implies the heterogeneity positively influenced pressure management because high injection rates, which may cause overpressure in the reservoirs, could be significantly reduced without substantially altering the total stored CO_2_ mass. We note that although chemical reactions are not considered in this study, the complex process of CO_2_-water-mineral interactions may play important roles on multiphase flow in subsurface reservoirs and trapping mechanisms.

Among the four potential trapping mechanisms, capillary trapping is a rapid and effective mechanism for rendering an injected fluid immobile and reducing the need to ensure caprock integrity. The capillary-trapping capacity of the conglomerate core investigated in this study was calculated using imbibition test results and the IR characteristic curve. While the Berea and Pohang sandstone cores showed approximately linear increases in capacity with initial CO_2_ saturation, the Janggi conglomerate displayed significant variations due to its highly heterogeneous porosity distribution. The conglomerate capacity ranged between 0.5 and 4.5%, depending on the initial saturation (0–0.4): for an initial saturation of 0.4, the conglomerate capacity ranged between 3–4%. Despite the fact that the trapping analyses showed lower overall capacities relative to homogeneous sandstone, the high porosity region of the conglomerate core displayed a similar capacity to sandstones, implying conglomerate formations could be an alternative for CO_2_ storage formation. CO_2_ injection and comprehensive monitoring in conglomerate formations will validate the feasibility of CO_2_ storage in the highly heterogeneous conglomerate formations.

## Methods

### Field site and geological setting

An onshore pilot-scale geologic CO_2_ storage project was launched in South Korea in 2011. The primary tasks (Phase I, 2012–2015) were the site-selection and initial characterization of potential pilot-scale storage sites. The Janggi conglomerate, composed of both clasts and silty sand matrix, was chosen as the target storage formation for the project. More recently, Phase II (2015–2017) was completed, including the drilling of seven boreholes to investigate the lithology of targeted and capping formations. The representative lithological log for this is illustrated in Fig. 1(a)^46^. The average porosity and permeability of the storage formation were 16.1% and 8.5 mD, respectively^47^. The overlying Seongdongri formation, composed of thick mudstone and massive dacitic tuffs, forms the regional sealing formation for CO_2_ storage, and a monitoring well (~40 m up-gradient from the injection well) was drilled. A comprehensive monitoring system was installed including a down-hole pressure and temperature measurements, vertical seismic profile (VSP), electrical resistivity tomography (ERT), distributed temperature sensing (DTS) and distributed acoustic sensing (DAS) along the well via an optical fiber, and U-tube downhole sampler.

### X-ray micro CT

The dimensions of the core-plug obtained from the Janggi conglomerate formation were 130 × 48 mm (length × diameter). X-ray computed tomography (CT), a non-destructive and non-invasive method, was used to investigate the spatial distribution of clasts within the silty sand matrix. The CT images used in this study were obtained at 140 kV and 200 μA. A total of 2,000 cross-sectional images were taken at 0.065 mm intervals with a micrometer-order pixel size. Subsequently, two-dimensional (2D) images were stacked to generate 3D core sample images.

### Multiphase flow tests with *in-situ* imaging technology

The apparatus for core-flooding experiments was designed to conduct multiphase flow tests. It was comprised of fluid (supercritical CO_2_ and brine) injection, core-holder, temperature controlling, confining, and back-pressure control systems (Supplementary Fig. S5). A 2D X-ray scanning system, consisting of an X-ray tube and a detector, was integrated into the core-flooding system in order to capture the real-time distribution of CO_2_ saturation distributions along the core sample. The detector board had two arrays of 128 channels each, corresponding to 256 detectors with a detector pitch of 0.4 mm. The array of the X-ray detector arrays were perpendicular to the core-axis, performing scans parallel to core-axis. The data output was in 16-bit format, ranging between 0 and 65,535. The produced 2D images were converted into CO_2_ saturations using the following equation: ${{\rm{S}}}_{{{\rm{CO}}}_{2}}=\,\frac{{{\rm{I}}}_{\exp }-{{\rm{I}}}_{{\rm{brinesat}}}}{{{\rm{I}}}_{{{\rm{CO}}}_{2}}-{{\rm{I}}}_{{\rm{brinesat}}}}$, where Ico_2_ and I_brinesat_ are the values of grayscale intensity obtained from the background scans of the core, which were saturated with CO_2_ and brine in the core, respectively. Finally, I_exp_ was the value obtained for the multiphase condition. The obtained CO_2_ saturation map may include noise mainly due to the relatively small density contrast between the two fluid phases (CO_2_ and brine) compared with the contrast between any one of the fluids and the rock^48^. In the experiments, NaI was used as a dopant to enhance the X-ray attenuation so that the CO_2_ saturation could be distinguished from the brine in the scanned image.

Two multiphase flow tests were conducted: (i) CO_2_ injection into a brine-saturated core, simulating the CO_2_ injection period (drainage) conditions; and (ii) brine injection following the CO_2_ injection test, reproducing post-injection period (imbibition) conditions. Downstream pressure was maintained during these two experiments at 10 MPa, and the temperature was set at 50 °C to replicate the subsurface environment at Janggi pilot site. Two different injection rates (q = 1.0 and 0.1 ml/min) were applied. The capillary numbers, ${{\rm{N}}}_{{\rm{c}}}=\,\frac{{\rm{H}}}{|{\rm{\Delta }}{P}_{c}|}\frac{{\rm{\Delta }}P}{L}$ (where L and H represent the length and diameter of the core, respectively; ${\rm{\Delta }}P$ represents the pressure drop; and $|{\rm{\Delta }}{P}_{c}|$ was calculated from the capillary pressure curves), were ~10^−1^ and ~10^−2^, for q = 1.0 and 0.1 ml/min, respectively.

### Pore size distribution and capillary pressure

The pore size distributions and capillary pressure curves for the silty sand matrix and clasts in the conglomerate sample were determined using a mercury injection porosimeter (MICP). To perform MICP tests, two small subsamples (~1 cm^3^) were taken from the conglomerate core each representing either the silty sand matrix or clasts. These subsamples were dried in a vacuum oven at 70 °C for 24 hours prior to testing. The MICP and corresponding mercury saturation measured in the mercury/air system were transformed into a capillary pressure and corresponding CO_2_ saturation for a CO_2_/brine system using the following relationship: $\frac{{P}_{c,C{O}_{2}}}{{P}_{c,Hg}}=\frac{{\sigma }_{C{O}_{2}}\,cos{\theta }_{C{O}_{2}}}{{\sigma }_{Hg}\,cos{\theta }_{Hg}}$, where ${P}_{c}$ (Pa) represents the capillary pressure, $\sigma$ (mN m^−1^) represents the interfacial tension (IFT) between two fluids, and $\theta$ (deg) represents the contact angle measured in the wetting phase. The IFT of ${\sigma }_{Hg}$ = 485 mN m^−1^ and ${\sigma }_{C{O}_{2}}$=32.6 mN m^−1^ was used in the conversion for the geologic CO_2_ sequestration conditions. The contact angles for the two systems were assumed to be equal^29^.

## Electronic supplementary material

Supplementary Information
