# Supplementary material for: Two-phase flow visualization under reservoir conditions for highly heterogeneous conglomerate rock: A core-scale study for geologic carbon storage
Source: Sci Rep. 2018 Mar 20;8:4869. doi: 10.1038/s41598-018-23224-6 (PMC5861079; doi:10.1038/s41598-018-23224-6)
Supplement: Supplementary file 1 — Supplementary Information [file 41598_2018_23224_MOESM1_ESM.pdf]

Supplementary Information

Two-phase flow visualization under reservoir conditions for highly heterogeneous conglomerate rock: A core-scale study for geologic carbon storage

Authors:

Kue-Young Kim [1,*], Junho Oh [2], Weon Shik Han [3], Kwon Gyu Park [1],
Young-Jae Shinn [1], Eungyu Park [2],

Affiliations:

[1] Korea Institute of Geoscience & Mineral Resources, Daejeon 34132, South Korea

[2] Dept. of Geology, Kyungpook National University, Daegu 41566, South Korea

[3] Dept. of Earth System Sciences, Yonsei University, Seoul 03722, South Korea

Corresponding author:

Kue-Young Kim (kykim@kigam.re.kr)

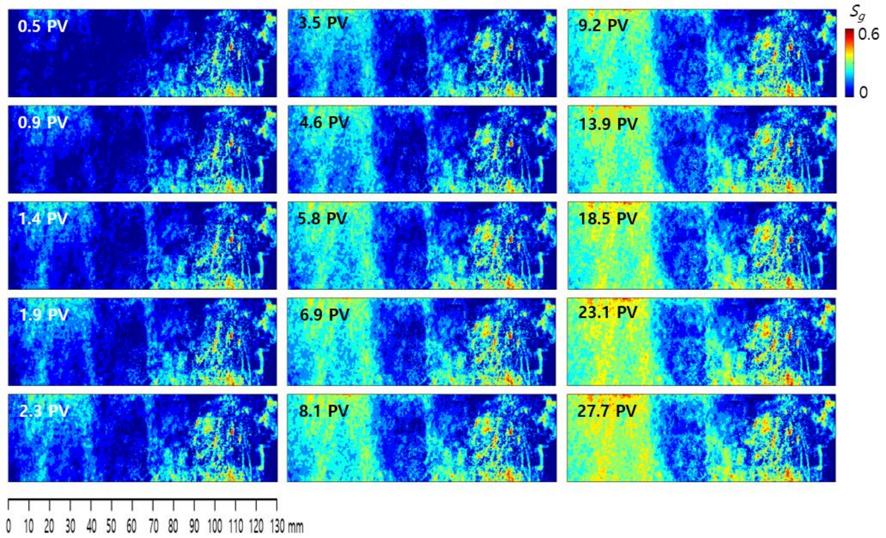

Figure S1. Snapshots of CO_2 saturation maps during the CO_2 injection tests (q = 0.1 ml/min) at different PVs.

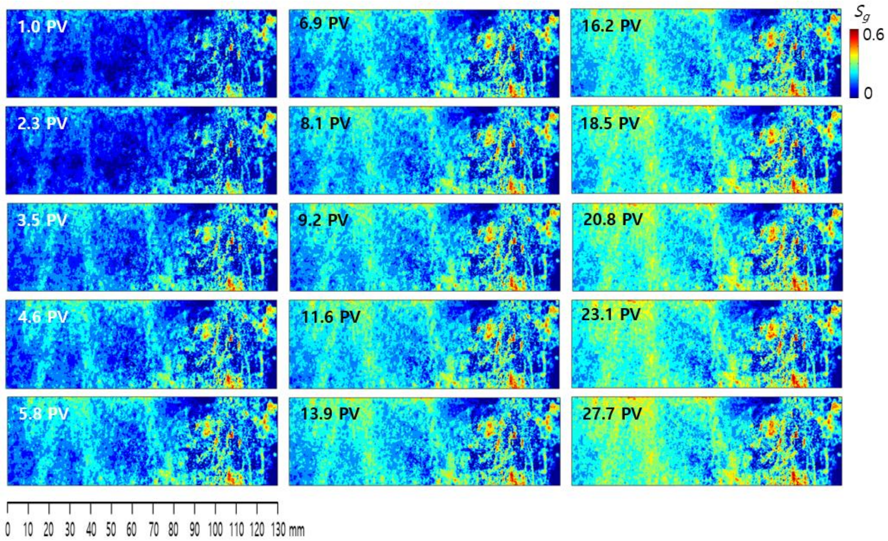

Figure S2. Snapshots of CO_2 saturation maps during the CO_2 injection tests (q = 1.0 ml/min) at different PVs.

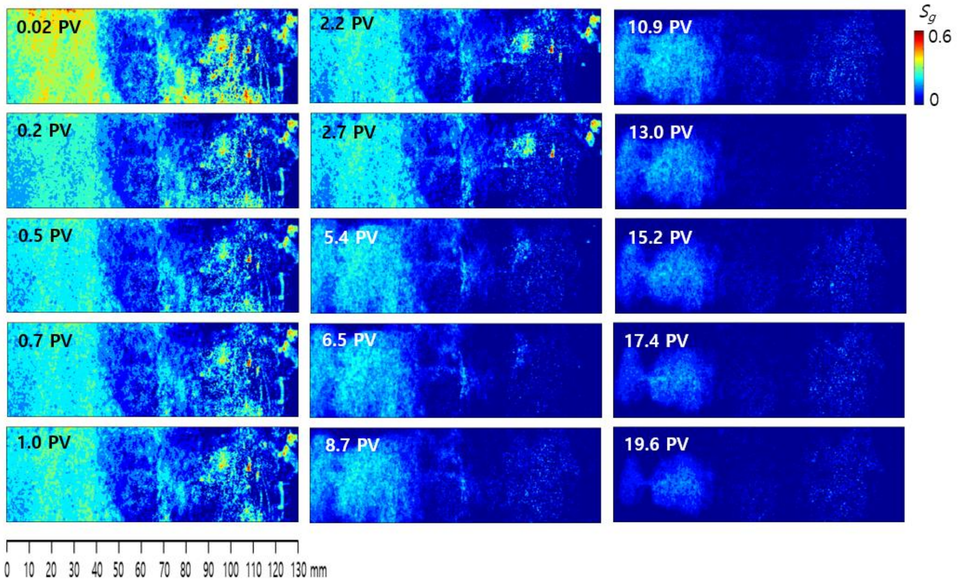

Figure S3. Snapshots of CO_2 saturation maps during water injection tests (q = 0.1 ml/min) at different PVs.

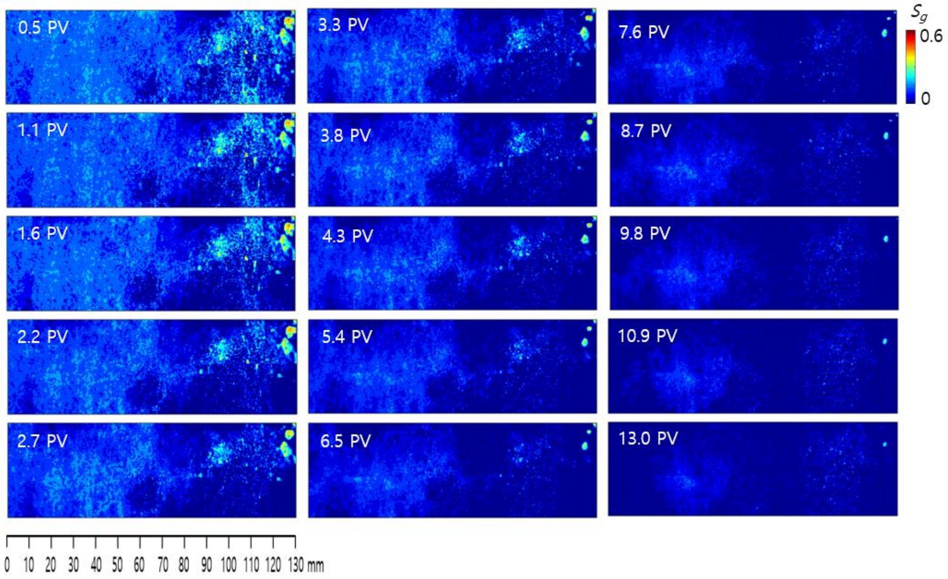

Figure S4. Snapshots of CO_2 saturation maps during water injection tests (q = 1.0 ml/min) at different PVs.

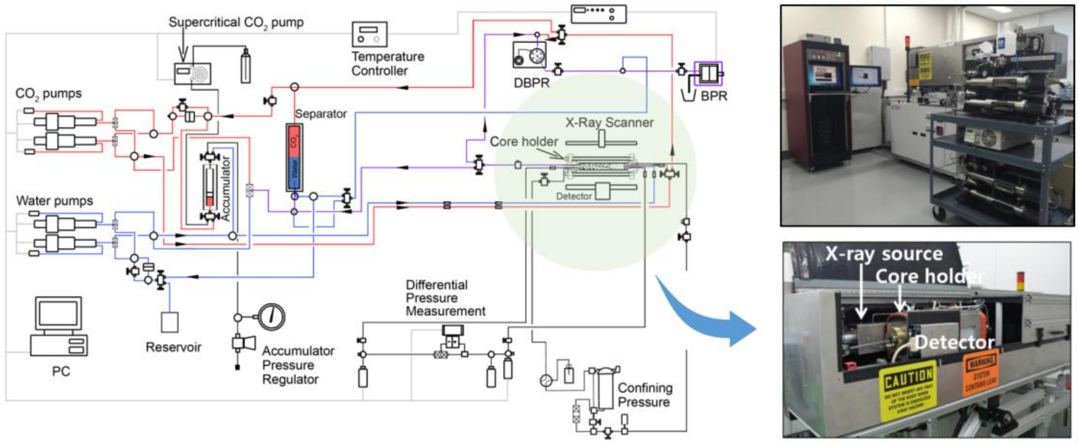

Figure S5. A schematic diagram of the core-flooding experimental setup. The upper right image shows the overall apparatus, and the lower right image shows the core-holder suited with X-ray tube and detector.

Table S1. Summary of core samples

	Janggi conglomerate	Pohang sandstone	Berea sandstone
Location	Janggi conglomerate was sampled from the target storage formation for on-shore pilot-scale CO_2 storage project in South Korea	Pohang sandstone was sampled from a testbed that was developed for CO_2 injection tests in Pohang basin in South Korea	Berea sandstone was obtained from Berea sandstone core company
Composition	Clasts and sandy matrix	Sand and silt	Homogeneous sand
Length (mm)	130	70	150
Diameter (mm)	48	38	38
Porosity (-)	variable	0.23	0.20
Permeability (mD)	5	23	170
